# Cortical Afferents and Myeloarchitecture Distinguish the Medial Intraparietal Area (MIP) from Neighboring Subdivisions of the Macaque Cortex

**DOI:** 10.1523/ENEURO.0344-17.2017

**Published:** 2017-12-08

**Authors:** Sophia Bakola, Lauretta Passarelli, Tony Huynh, Daniele Impieri, Katrina H. Worthy, Patrizia Fattori, Claudio Galletti, Kathleen J. Burman, Marcello G. P. Rosa

**Affiliations:** 1 Biomedicine Discovery Institute and Department of Physiology, Monash University, Clayton, Victoria 3800, Australia; 2 Australian Research Council, Centre of Excellence for Integrative Brain Function, Monash University Node, Clayton, Victoria 3800, Australia; 3 Department of Pharmacy and Biotechnology, University of Bologna, Bologna, 40126, Italy

**Keywords:** Connectivity, grasping, parietal, primate, reaching, visuomotor integration

## Abstract

The parietal reach region (PRR) in the medial bank of the macaque intraparietal sulcus has been a subject of considerable interest in research aimed at the development of brain-controlled prosthetic arms, but its anatomical organization remains poorly characterized. We examined the anatomical organization of the putative PRR territory based on myeloarchitecture and retrograde tracer injections. We found that the medial bank includes three areas: an extension of the dorsal subdivision of V6A (V6Ad), the medial intraparietal area (MIP), and a subdivision of area PE (PEip). Analysis of corticocortical connections revealed that both V6Ad and MIP receive inputs from visual area V6; the ventral subdivision of V6A (V6Av); medial (PGm, 31), superior (PEc), and inferior (PFG/PF) parietal association areas; and intraparietal areas AIP and VIP. They also receive long-range projections from the superior temporal sulcus (MST, TPO), cingulate area 23, and the dorsocaudal (area F2) and ventral (areas F4/F5) premotor areas. In comparison with V6Ad, MIP receives denser input from somatosensory areas, the primary motor cortex, and the medial motor fields, as well as from visual cortex in the ventral precuneate cortex and frontal regions associated with oculomotor guidance. Unlike MIP, V6Ad receives stronger visual input, from the caudal inferior parietal cortex (PG/Opt) and V6Av, whereas PEip shows marked emphasis on anterior parietal, primary motor, and ventral premotor connections. These anatomical results suggest that MIP and V6A have complementary roles in sensorimotor behavior, with MIP more directly involved in movement planning and execution in comparison with V6A.

## Visual Abstract

**Figure:**
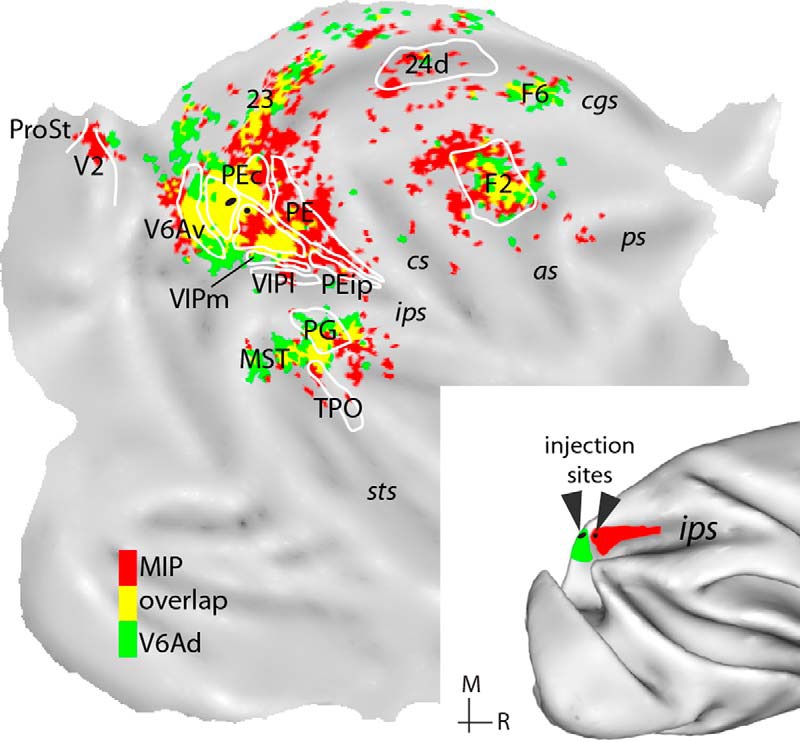

## Significance Statement

The medial bank of the intraparietal sulcus encompasses a parietal reach region (PRR) where neurons are involved in the planning of visually guided arm movements, which has been the subject of interest in research related to prosthetic arm control. To clarify the anatomic subdivisions of PRR, we examined the connections of different sites within and rostral to this region with other areas of the macaque cortex. Based on differences in the density and modality specificity of connections and histologic characteristics, we propose a subdivision of the medial bank into areas. This anatomic scheme, which incorporates features of previous proposals but refines the boundaries, may help guide future studies aimed at clarifying the functions of different medial intraparietal areas.

## Introduction

The cortex in the medial bank of the macaque intraparietal sulcus, originally described as part of Brodmann’s area 5, has been the subject of several studies, which attempted to map its subdivisions based on histologic features, connections and sensory representations (Sakata et al. 1973; Mountcastle et al. 1975; Pandya and Seltzer, 1982; Pons et al. 1985; Colby et al. 1988; Iwamura, 2000; Lewis and Van Essen, 2000a; Seelke et al. 2012; Mayer et al. 2016). According to most current studies, the caudal part of the medial bank includes a medial intraparietal area (MIP), first defined on the basis of myeloarchitecture and connections with extrastriate cortex (Colby et al. 1988). Caudal to MIP is area V6A, which is typically depicted as being located within and around the parieto-occipital sulcus, slightly invading the medial bank of the intraparietal sulcus (Luppino et al. 2005). However, the anatomic criteria that differentiate MIP from surrounding cortex have not been explored in detail.

At the same time, the medial bank of the intraparietal sulcus and the anterior parieto-occipital sulcus have been the focus of numerous studies related to the planning and guidance of arm movements (e.g., Colby and Duhamel, 1991; Johnson et al. 1996; Fattori et al. 2001; Battaglia-Mayer et al. 2003; Gregoriou and Savaki, 2003; Hadjidimitrakis et al. 2014b). A wide region, which likely includes parts of V6A and MIP, is often referred to as the parietal reach region (PRR; Snyder et al. 1997, 1998; Andersen et al. 2014a). Neurons in this region also display activity related to other aspects of visuomotor integration, such as eye movements (Snyder et al. 1997; Breveglieri et al. 2012; Hadjidimitrakis et al. 2012). Some evidence for functionally distinct sectors in the medial bank has emerged, based, for example, on descriptions of variability in deficits after permanent or reversible cortical lesions (Rushworth et al. 1997; Battaglini et al. 2002; Padberg et al. 2010; Hwang et al. 2012; Yttri et al. 2014). However, the relationship between sites related to these functions and anatomically defined areas has remained difficult to ascertain. In part, this is due to the use of different terminologies by research groups. More fundamentally, however, the anatomic organization of the medial bank of the intraparietal sulcus has not been addressed in sufficient detail. Previous studies in macaques have explored the cortical connectivity of other posterior parietal areas (Cavada and Goldman-Rakic, 1989a,b; Lewis and Van Essen, 2000b; Marconi et al. 2001; Morecraft et al. 2004; Gamberini et al. 2009; Bakola et al. 2010, 2013; Passarelli et al. 2011, 2017), but studies that did target the medial bank (Pandya and Seltzer, 1982; Blatt et al. 1990; Prevosto et al. 2011) included relatively few tracer injections, precluding comparisons of results obtained in different locations.

In the present study, we examined the afferent cortical connections and histology of the medial bank of the intraparietal sulcus in macaques. Based on these anatomic features, we propose a tripartite subdivision, which, while incorporating many features of previous proposals, refines the areal boundaries. This anatomic scheme, which provides a firm basis for subdivision of the PRR into two areas (V6A and MIP), may help guide future functional studies.

## Materials and Methods

Fluorescent tracers were injected in the medial bank of the intraparietal sulcus in six macaque monkeys (*Macaca fascicularis* and *M. nemestrina*; Table 1). Some of these animals received additional tracer injections (not reported here) or were also studied in acute sessions of electrophysiological recordings under anesthesia. Experimental protocols were approved by the Monash University Animal Experimentation Ethics Committee and the Bioethical Committee of the University of Bologna and were updated during the project according to the most recent institutional regulations. All procedures followed the guidelines of the Australian Code of Practice for the Care and Use of Animals for Scientific Purposes and the European Union Directives 86/609/EEC and 2010/63/EU on the care and use of laboratory animals.

**Table 1.** Summary of experimental cases

Case	Designation	Species/sex	Weight (kg)	Injection site	Amount (µl), concentration (%)
1	MF7-DY	*M. fascicularis/*M	3.7	V6Ad	0.3, 1.5
2	NF228-DY	*M. nemestrina*/F	3.0	V6Ad	0.25, 1.5
3	MF7-FR			MIP	0.5, 15
4	NM31-FB	*M. nemestrina*/M	7.8	MIP	0.35μl, 1.5
5	MF7-FB			MIP	0.3, 1.5
6	A9-CTB	*M. fascicularis*/M	4.1	MIP	2, 1
7	MF8-FE	*M. fascicularis*/M	5.3	MIP	1, 15
8	MF7-FE			MIP/PE	0.7, 15
9	MF10-DY	*M. fascicularis*/M	3.6	PEip	0.25, 1.5

CTB, cholera toxin subunit B, conjugated with Alexa 488; DY, diamidino yellow; FB, fast blue; FE, fluoroemerald MW 10,000; FR, fluororuby MW 10,000. For FE and FR, only retrograde labeling is reported here.

### Surgical procedures and tissue processing

Surgeries took place in standard aseptic conditions, and in all cases the heart rate, blood pressure, respiratory depth, and body temperature were continuously monitored. Animal A9 was pretreated with injections of atropine (0.04 mg/kg, i.m.) and ketamine hydrochloride (15 mg/kg, i.m.) and, after 30 min, anesthetized with sodium thiopental (8 mg/kg, iv), with additional doses administered as required. The other animals were pretreated with i.m. injections of diazepam (1.0 mg/kg) and atropine (0.04 mg/kg); anesthesia was induced 30 min later with a ketamine/Dormitor/butorphanol cocktail (0.1 mg/kg i.m.), after which the animals were intubated and maintained with isoflurane (0.5%–2%). Hydration was provided by constant iv infusion of Hartmann’s solution. Dexamethasone (0.3 mg/kg, i.m.) and Norocillin (25 mg/kg, i.m.) were also administered at the start of the procedures.

The animals were secured on the stereotaxic apparatus, and craniotomies were performed over the posterior parietal cortex to reveal the intraparietal sulcus. Injection sites were selected by direct visualization of the sulcal geometry and were later assigned to architectonic subdivisions after histologic examination of postmortem material. Fluorescent tracers (Table 1) were injected using a microsyringe that had a glass micropipette attached to its needle. After the injection procedures, the cortical surface was covered with Gelfilm, the bone flap was fixed back in place with dental acrylic, and the muscles and skin were sutured. On recovery from anesthesia, the animals were returned to their home cages and closely monitored. For the following 2–3 d, the animals were maintained on analgesics (A9: Ketorolac, 1 mg/kg, i.m.; other cases: carprofen 4 mg/kg, s.c., or Temgesic 0.01 mg/kg, i.m.), and antibiotics (erythromycin, 1–15 mL/10 kg, or norocillin, 0.17 mL/kg).

After a survival period of 14 d, the animals were premedicated as above before receiving a lethal injection of sodium thiopental or pentobarbitone (100 mg/kg, iv). They were first perfused with heparinized saline or phosphate buffer, and then with 4% paraformaldehyde in 0.1 M phosphate buffer at pH 7.4. Case A9 was subsequently perfused with 4 liters of 5% glycerol in the same buffer. The brains were removed from the skull, photographed, cryoprotected by immersion in buffered solutions of 10% and 20% glycerol (A9) or sucrose (10%–30%, other cases) until they sank, and then snap-frozen and stored at –80°C. Sections of 50 or 60 µm were cut in the coronal plane, using a freezing microtome (A9) or a cryostat (other cases). Every fifth section was left unstained for observation under the fluorescence microscope, and adjacent series were stained for Nissl substance and for myelin with the Gallyas method (Gallyas, 1979). All sections were coverslipped with DPX, after rapid dehydration in ethanol and clearing with xylene.

### Data analysis

Neurons labeled with fluorescent tracers were visualized using a Zeiss Axioskop microscope equipped with 10× and 20× dry objectives. For all sections examined, the pial and inner boundaries of the cerebral cortex, the outlines of the injection sites, and the location of labeled cells were charted using software tools that read the input of *X*/*Y* transducers mounted on the microscope stage. Digital reconstructions of the cortical surface were generated with CARET software (http://www.nitrc.org/projects/caret/, Van Essen et al. 2001), from midthickness section contours, as described previously (Galletti et al. 2005; Gamberini et al. 2009). The same software was used to prepare the density maps of labeled neurons by projecting the location of each neuron to the nearest midthickness contour of the 3D reconstruction (Bakola et al. 2010; Passarelli et al. 2011). A quantitative measure of the strength of projections from various cortical regions is reported as the percentage of labeled cells per total number of labeled cells in each case (Table 2).

**Table 2.** Percentages of extrinsic projections (% of total) in various cortical areas after injections in the medial bank of the intraparietal sulcus

Injected area	V6Ad		MIP					MIP/PE	PEip
Case	1	2	3	4	5	6	7	8	9
Extrastriate (EXT)									
V6	*	3.2	*	0.6	3.4	0.9	*	*	
Other EXT	0.9	*	2.2	*	5.7	0.7			
Medial parietal									
V6Av	32.0	43.4	6.9	6.8	12.1	3.5	9.8	*	*
V6Ad	^#^	^#^	33.0	19.7	21.1	7.9	11.4	3.7	
PGm	3.2	0.6	*	3.1	*	*		*	*
PEci/31	3.0	4.2	3.2	11.3	7.8	*		7.7	*
Superior parietal (SPL)									
PE	0.5	*	8.1	9.5	13.0	5.3	17.4	49.4	21.0
PEip	*	*	1.6	2.7	4.2	9.5	10.2	6.1	^#^
PEc	22.6	3.2	31.0	15.0	13.2	3.0	2.7	10.6	6.5
Other SPL	*		0.5	*	*	1.4	*	3.4	28.1
Intraparietal IPS									
MIP	15.7	20.0	^#^	^#^	^#^	^#^	^#^	^#^+	5.8
VIP	*	11.2	*	4.1	4.1	12.9	11.3	1.3	9.0
LIP	*	*	*	*	0.8	1.2	*		
AIP	*	1.1	*	*	*	4.1	1.4	*	1.8
Inferior parietal (IPL)									
Opt/PG	5.9	2.4	*	*	*	1.1	*	*	
PFG/PF	1.0	3.1	*	*	*	2.4	0.5	*	*
ParOp	*	*	1.2	0.7	1.3	3.1	3.3	1.1	3.3
Temporal									
cST	6.4	1.0	1.9	3.5	4.5	5.7	1.3	*	*
Other Temporal		*	*	*	*	*			*
Limbic									
23	0.8	0.5	*	1.5	*	1.8	*	*	*
24	*	*	*	3.0	*	4.0	1.1	0.8	2.7
Rs	*	*	*	*	*	*	*		
Motor/premotor									
F1	*	*	0.9	1.3	1.1	2.3	10.0	3.9	16.6
F2	5.1	3.3	6.0	10.0	5.0	19.3	16.5	9.1	*
F3	*	*	0.8	1.2	0.6	*	0.9	0.9	0.6
F5/F4		0.5	*	1.2	*	5.2	1.0	*	1.5
F7	*	*	*	1.1	*	*	*	*	
F6	0.8	0.5	*	1.0	*	*	*	*	
Prefrontal (PrFr)									
SEM/FEF		*	*	2.2	*	3.2			1.4
Other PrFr		*	*	*	*	*	*	*	
*n*	7276	19,235	4369	15,202	22,129	6808	1239	2230	6457

^#^Location of injection site; *<0.5% of total projections; +, injection site invaded area PE; *n,* number of extrinsically labeled cells. “Other EXT” includes combined percentages of labeled neurons in V2, Vis, area prostriata, V4, and DP.

To examine the consistency in the pattern of distribution of label across cases, we used the Kendall coefficient of concordance (*W*, evaluated by χ^2^), a nonparametric statistical measure employed previously in anatomic studies (Bakola et al. 2013; for detailed discussion, Reser et al. 2013; Burman et al. 2014a,b). Data from the two injections in V6Ad were compared with the Spearman rank correlation (*Rs*); as described before (Legendre, 2005), for pairwise correlations, *W* is a linear transformation of *Rs*. For the present analysis, we grouped projections from different source areas into nine cortical sectors (Table 2), to correct for low or zero cell counts.

### Identification of cortical areas containing extrinsic labeled cells

The nomenclature and boundaries of the cortical areas that contained labeled cells after injections in the medial intraparietal region were based on published criteria or relative to sulcal landmarks, using previous published maps as a guide.

#### Posterior parietal cortex

The architectonic criteria of Pandya and Seltzer (1982) were used to subdivide the superior parietal lobule into areas PE and PEc. The inferior parietal lobule was subdivided according to Pandya and Seltzer (1982), Andersen et al. (1990), and Gregoriou et al. (2006). Area LIP in the lateral intraparietal sulcus was identified based on descriptions by Blatt et al. (1990) and Medalla and Barbas (2006). The fundus of the intraparietal sulcus is occupied by area VIP (Colby et al. 1993); in myelin-stained tissue, we identified medial and lateral subdivisions (VIPm, VIPl; Lewis and Van Essen, 2000a), but for analysis, these were grouped under the term VIP. We recognized parieto-occipital area V6 (largely coextensive with area PO; Colby et al. 1988; Galletti et al. 2005) on myeloarchitectonic grounds (Luppino et al. 2005).

#### Temporal lobe

We used the collective term cST for dorsal parts of the caudal superior temporal sulcus, including areas MST and caudal TPO (TPOc); although the region has been reported to contain distinct architectonic patterns (Desimone and Ungerleider, 1986; Boussaoud et al. 1990; Lewis and Van Essen, 2000a), we could not consistently identify these across animals.

#### Mesial surface

The subdivision of areas on the medial wall and cingulate sulcus (PGm, 23, 24) were based on definitions by Matelli et al. (1991), Kobayashi and Amaral (2000), Morecraft et al. (2004), Vogt et al. (2005), and Passarelli et al. (2017). Difficulties in identifying areas PEci and 31 in the caudal cingulate sulcus led us to assign a collective area 31/PEci designation for labeled cells in that part of cortex. Ventromedial parts of the precuneate cortex (ventral to area PGm) have been tentatively designated “Vis” (Kobayashi and Amaral, 2000). Further ventrally, labeled cells in rostral parts of the dorsal calcarine sulcus were attributed to the second visual area (V2; (Gattass et al. 1981; Rosa et al. 1988) and area prostriata (Yu et al. 2012).

#### Frontal lobe

The frontal motor and premotor cortices were subdivided into areas F1–F7 according to the criteria of Matelli et al. (1991) and Belmalih et al. (2007). We used the term “SEM” for the territory in ventral parts of the posterior bank and depths of the arcuate sulcus, which contains the macaque smooth pursuit eye field (Stanton et al. 2005). Labeled cells in the anterior bank and convexity of the arcuate sulcus were allocated to the frontal eye fields (8/FEF; Moschovakis et al. 2004; Gerbella et al. 2010). The few labeled cells in the dorsolateral prefrontal cortex, near and in the principal sulcus, were attributed to areas 9/46 (Petrides and Pandya, 1999).

## Results

Here we describe the pattern of cortical projections to the medial bank of the intraparietal sulcus and adjacent rostral parieto-occipital sulcus, based on data from nine fluorescent tracer injections in six macaques. As summarized in Table 1, we have assigned six of these injections to area MIP and two to the dorsal part of area V6A (V6Ad). The pattern of connections of V6Ad has been previously described in detail (Gamberini et al. 2009); in the present study, data from two new cases will be used to contrast this connectivity with that of MIP, which is located more rostrally along the medial wall of the intraparietal sulcus. One additional case (case 9) illustrates the connection pattern of the medial bank beyond the rostral border of MIP.

### Identification of medial intraparietal sulcus areas

**Figure 1.**
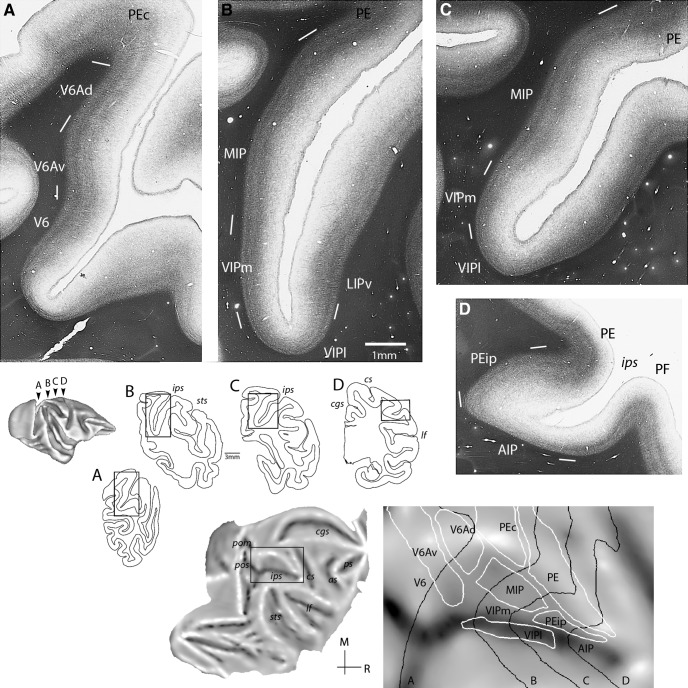
Top: Myeloarchitecture of the medial intraparietal region shown in four coronal sections (***A–D***) of a representative case (case 7). White lines mark uncertainties in the definition of areal boundaries. Areas V6A (***A***) and MIP (***B***, ***C***) were characterized by well-separated bands of Baillarger, with V6A showing a thicker inner band and overall radial organization; rostral area PEip was identified by relatively light myelination (***D***). Bottom: Boundaries (in white) of medial intraparietal and neighboring divisions projected on a flat map. Midthickness contours of sections ***A–D*** are shown in black lines. In this and following unfolded maps, the gray shading represents cortical curvature: convex surfaces (e.g., “lips” of sulci) appear lighter, whereas concave surfaces (e.g., banks of sulci) are darker. AIP, anterior intraparietal subdivision; LIPv, ventral subdivision of LIP; MIP, medial intraparietal area; PE, PEip, subdivisions of area 5; PF, subdivision of inferior parietal cortex; V6Ad, V6Av, dorsal and ventral subdivisions of area V6A; VIPl, VIPm, lateral and medial subdivisions of area VIP; sulci: *as*, arcuate; *cgs*, cingulate; *cs*, central; *ips*, intraparietal; *lf*, lateral fissure; *pom*, medial parieto-occipital; *pos*, parieto-occipital; *ps*, principal; *sts*, superior temporal. M, medial; R, rostral.

The following descriptions are based on low-power views of myelin-stained sections, which, in our experience, proved the most useful for areas in the medial intraparietal cortex [in agreement with Lewis and Van Essen (2000a)]. Fig. 1*A–D* highlights the architectonic transitions identified in the present study. The same figure illustrates, on a flat map, the corresponding midthickness section contours and areal boundaries of a representative case (case 7). In this and the following maps, architectonic borders illustrate the core of delineated areas (or zones), as assessed by histologic criteria; uncertainties in the definition of borders, for example, in Fig. 1*A–D*, are marked by white lines.

The medial intraparietal cortex is moderately myelinated and is characterized by the presence of two distinct bands of Baillarger. In a caudal-to-rostral sequence (Fig. 1*A–D*), we recognized three architectonic variations: areas V6A, MIP, and a subdivision of area PE (PEip). The most caudal pattern (V6A) had a relatively thick inner band and generally radial orientation (Fig. 1*A*), whereas MIP was characterized by a thinner inner band and more matted appearance (Fig. 1*B*, *C*). Dorsal and ventral subdivisions of V6A (V6Ad, V6Av) were distinguished in our material as progressions in myelin staining (Fig. 1*A*), as per earlier descriptions (Luppino et al. 2005); according to previous functional studies, these are best seen as subdivisions of a single area, V6A, rather than separate areas (Gamberini et al. 2011).

In more rostral parts of the medial bank (approximately at the coronal level corresponding to the dorsal tip of the central sulcus; Fig. 1*D*), the myelin density becomes lighter and the bands of Baillarger become less easily discerned. We termed this region, which falls within the architectural designation of area 5 (Lewis and Van Essen 2000a), as PEip, noting that it comprises only a subset of the original larger portion of the medial bank forming corticospinal connections (Matelli et al. 1998).

The adjacent cortex near the fundus, and continuing into the lateral bank, has been previously designated as the anterior intraparietal area, AIP (Preuss and Goldman-Rakic, 1991; Lewis and Van Essen, 2000a). However, the same term has been employed by physiologic (Sakata et al. 1995; Murata et al. 2000) and connectional (Borra et al. 2008) studies that targeted rostral parts of the lateral bank of the intraparietal sulcus, in relation to grasping manipulations. These conflicting definitions of AIP differ in their connectivity profiles (Lewis and Van Essen, 2000b), but a comparative anatomic study is still lacking. Pending further investigations, we retained the term AIP for rostral parts around the fundus and in the lateral bank of the intraparietal sulcus.

**Figure 2.**
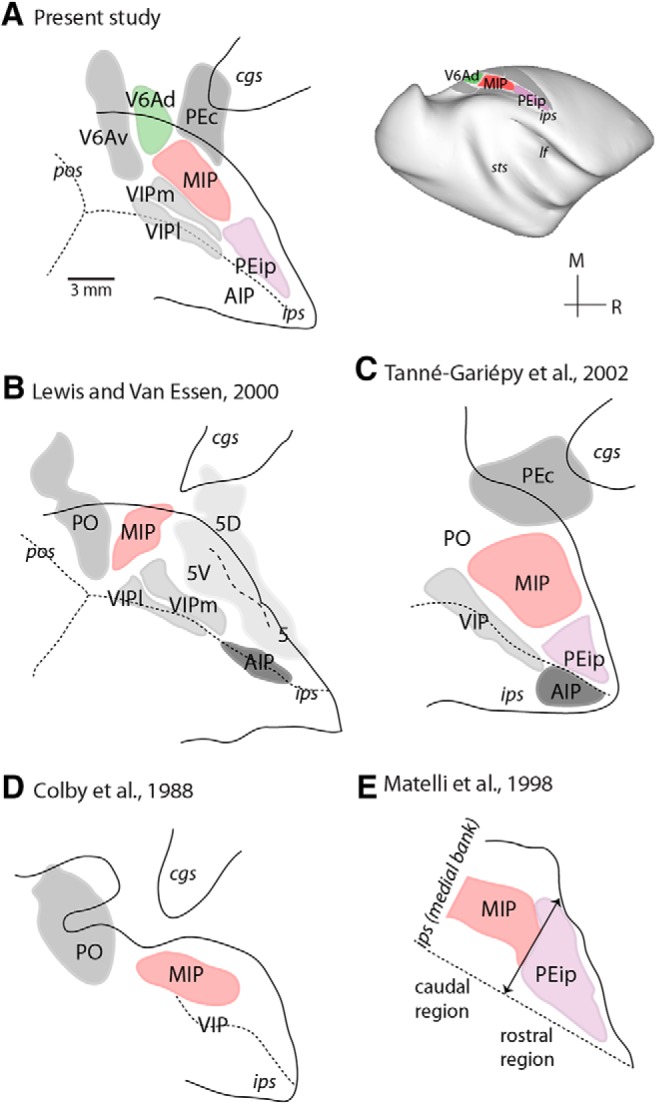
Architectonic divisions of the medial intraparietal and adjacent regions by different authors (***A–E***). Solid lines, outlines of unfolded sulci; dotted lines, fundus of sulci (medial bank of the intraparietal sulcus on top); dashed line, division between parietal areas 5D and 5V. PO, parieto-occipital area. Top right: 3D reconstruction of a macaque brain illustrating parietal subdivisions identified in the present study.

Comparison of this partitioning scheme with that proposed by Lewis and Van Essen (2000a) suggests that the observed differences are a reflection of the chosen terminology (Fig. 2). In particular, architectural field V6Ad of the present nomenclature appears to partially overlap with field MIP of the earlier study, whereas the presently defined MIP substantially overlaps with field 5V. The cortical territory assigned to MIP in the present study also overlaps, at least partially, with area PEa of previous proposals (Pandya and Seltzer, 1982; Morecraft et al. 2004). Overall, the present partitioning scheme appears more similar to that put forward earlier by Tanné-Gariépy et al. (2002). We recognize that the use of multiple terminologies assigned to overlapping cortical regions could confound the interpretation of results, but considering the much more extensive current information about the anatomy and physiology of V6A (Gamberini et al. 2009, 2011; Passarelli et al. 2011), the original definition of area MIP by Colby et al. (1988), and the results of tracer injections (see below), we believe that the present nomenclature provides an accurate synthesis of current knowledge.

### Overview of connections

**Figure 3.**
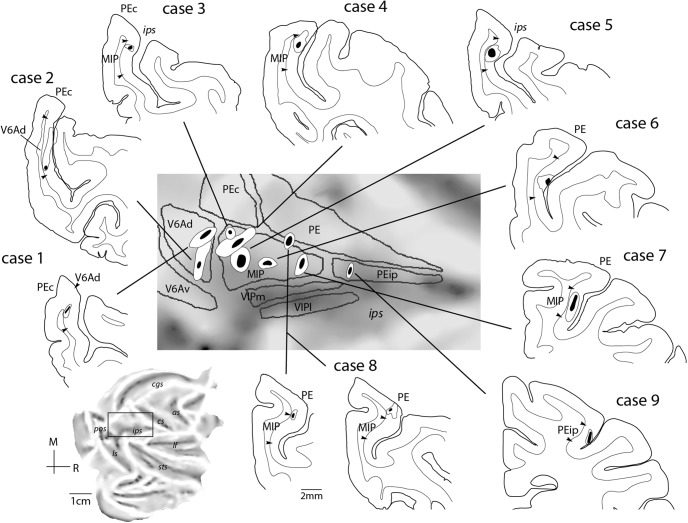
Summary of locations of injection sites. Coronal sections showing the centers (black) and halos (white) of injection sites for each of the cases presented in this study. The boundaries of areas are also shown. A summary of the injection sites for V6Ad (cases 1 and 2), MIP (cases 3–8), and PEip (case 9) is presented on the unfolded reconstruction of a macaque left hemisphere (expanded view in the center). Other abbreviations as in Fig. 1.

Fig. 3 reports the locations of the nine injection sites included in the present study, shown in coronal sections. To facilitate orientation, the estimated locations are projected on the surface of a representative “unfolded” macaque hemisphere, together with the boundaries of posterior parietal areas (Fig. 3, middle panel). The quantitative findings from individual cases are reported in Table 2. For the purposes of a summary, in the table we have combined regions that contained few labeled cells into groups based on anatomic location or functional similarities. In the following sections, we report on the corticocortical connections of the above myeloarchitectural fields in the medial bank of the intraparietal sulcus, from caudal to rostral, with the focus on identifying their shared and distinguishing patterns of connections.

#### V6Ad connections

**Figure 4.**
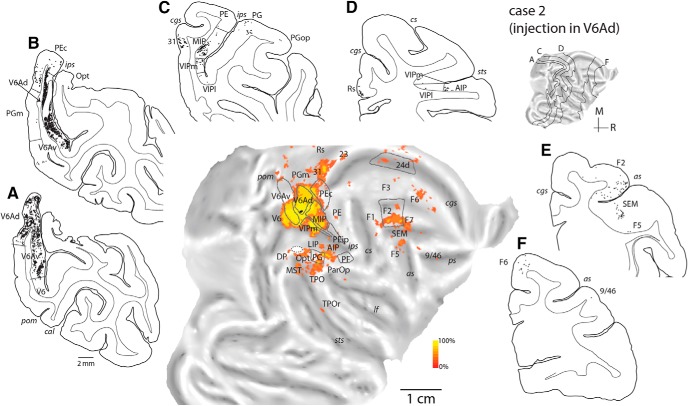
Cortical distribution of retrogradely labeled cells in case 2 (NF228-DY, injection in V6Ad). ***A–F***, Coronal sections were taken at the levels indicated on the flat map of the reconstructed hemisphere. Each black point represents a labeled cell. The quantitative distribution of labeled cells is shown on the two-dimensional reconstruction of the brain (middle panel). Boundaries for major architectonic divisions are reported on the series of coronal sections and on the flat map (gray lines). In the flat map, the location of the center of the injection site is shown in black. *cal*, calcarine sulcus. Other abbreviations as in Fig. 1.

Two injections were placed in the caudalmost parts of the intraparietal sulcus, near the location where this sulcus merges with the anterior bank of the parieto-occipital sulcus (cases 1 and 2; Fig. 3). These injections were within the currently recognized borders of area V6A (subdivision V6Ad), an assessment that was supported by application of the myeloarchitectural criteria of Luppino et al. (2005) and by the consistency in the pattern of projections (*Rs* = 0.733, df = 7, *p* = 0.02). The injection in case 1 was near the dorsal border of V6Ad with superior parietal lobule area PEc, whereas that in case 2 was located near the ventral border of V6Ad with V6Av. In case 2, there was a minor spill of tracer in the dorsal part of the lateral bank of the intraparietal sulcus (area LIP, Fig. 4B; white oval on the flat map of Fig. 4). However, we observed no evidence of the long-range transport typical of LIP, as shown by the lack of labeled neurons in the middle temporal area, MT, and the temporal area TEO (Blatt et al. 1990). Fig. 4 illustrates the distribution of retrograde label in case 2 in representative coronal sections and an unfolded view of the reconstructed cortical surface; a comparison of the connectional patterns after V6A (case 1) and MIP (case 3) injections is shown in Fig. 6*A*.

Both injections in V6Ad revealed substantial numbers of labeled neurons in V6Av (Fig. 4*A*, *B*) and, rostrally, in MIP (Fig. 4*C*), in addition to moderate input from the caudal (PG/Opt, Fig. 4*B*, C) and rostral (PFG) cytoarchitectural areas of the inferior parietal lobule. Much weaker parietal lobe projections originated in the dorsal parietal convexity (area PE), and in lateral intraparietal areas LIP and AIP (Fig. 4*D*). Label from area PEc (Fig. 4*B*) followed a dorsoventral trend; it was strong in case 1, but weak in case 2 (Table 2). The reverse trend was observed with respect to the connections of area VIP (primarily the medial subdivision, VIPm; Fig. 4*C*, *D*) along the fundus of the intraparietal sulcus (Table 2). Finally, the lateral somatosensory association areas [PGop, Fig. 4*C*, and Ri; grouped under parietal operculum (ParOp) in Table 2 and on the unfolded maps] contained a small number of neurons in both cases.

In the temporal lobe, both cases revealed relatively sparse but consistent projections from the superior temporal sulcus areas MST and TPOc [Fig. 4; in Table 2, these appear grouped under caudal superior temporal (cST)]. In addition, the rostral sector of polysensory area TPO (TPOr; Lewis and Van Essen, 2000a; Fig. 3*B*) included labeled neurons in case 2. Further caudally, visual cortex connections were evidenced by projections from area V6 (Fig. 4*A*) and in the dorsal part of the prelunate cortex (dorsal prelunate area, DP).

The mesial surface of the brain revealed moderate to low numbers of labeled cells in area PGm (e.g., Fig. 4*B*), in subdivisions of caudal (areas 31/PEci, Fig. 4*C*; 23, Fig. 4) and rostral (area 24; Fig. 4) cingulate cortex, and in the retrosplenial region (Rs; areas 29 and 30; Fig. 4*D*). The injection in case 1 resulted in sparse labeled cells in the ventromedial cortex near visual area V2 (Vis; Fig. 6*A*).

In the frontal cortex, moderate projections originated in dorsocaudal premotor area F2 (Fig. 4*E*), with smaller numbers of labeled neurons observed in other premotor subdivisions (F7, F5, F3/SMA, F6/preSMA; Figs. 4 and 6) and in the primary motor cortex (F1; Fig. 4). Other frontal lobe areas were devoid of label, except for a few neurons near the fundus of the dorsal branch of the arcuate sulcus (putatively in SEM; Fig. 4*E*) and in the dorsal periprincipal region (caudal area 9/46, case 2; Fig. 4*F*).

The pattern of label we observed after injections in lateral (caudal intraparietal) parts of V6Ad followed the general connectivity trend for this area, observed in an earlier study in which injections were located in the parieto-occipital sulcus and on the mesial surface (Gamberini et al. 2009), confirming characteristic input from parietal, dorsal premotor, and caudo-dorsal temporal regions. The few differences between the present and previous study were mainly quantitative: projections from the ventral parieto-occipital cortex (V6Av and V6) were somewhat denser than in the previous report, whereas those from the prefrontal cortex were less substantial.

#### MIP connections

**Figure 5.**
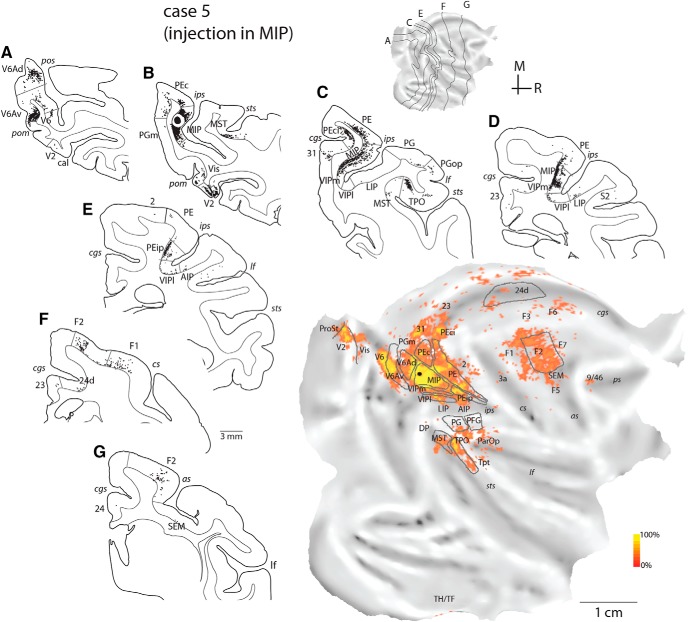
Cortical distribution of retrogradely labeled cells in case 5 (MF7-FB, injection in MIP). ***A–G***, Coronal sections were taken at the levels indicated on the flat map of the reconstructed hemisphere. The quantitative distribution of labeled cells is shown on the two-dimensional reconstruction of the brain (bottom right). Other abbreviations as in Figs. 1 and 4.

**Figure 6.**
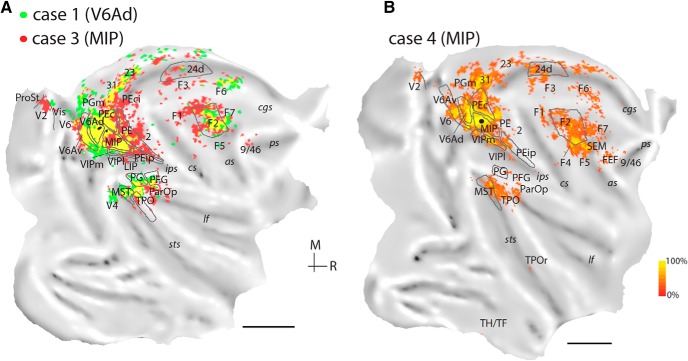
***A***, Comparison of the topographical distribution of label for V6Ad (MF7-DY, case 1, green) and MIP (MF7-FR, case 3, red) on the same hemisphere. Shared projection fields are shown in yellow. ***B***, Distribution of labeled cells after an injection in MIP in case 4 (NM31-FB). Scale bars = 1 cm. Other abbreviations as in Fig. 1.

**Figure 7.**
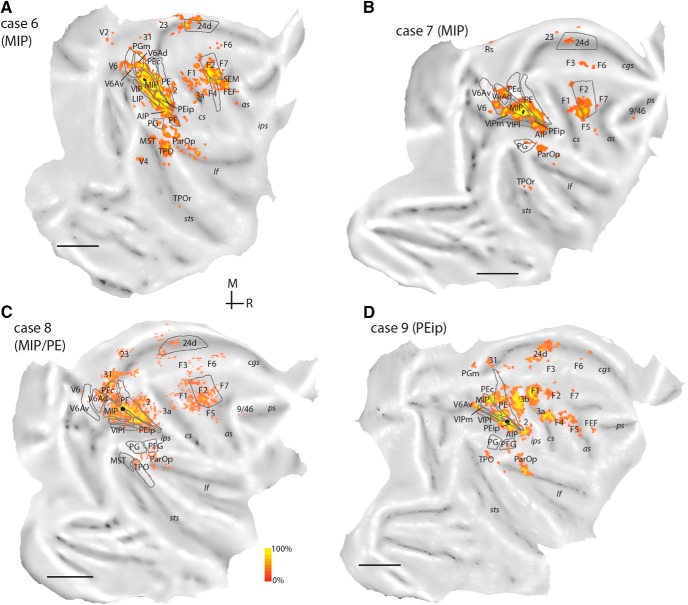
Distribution of labeled cells after injections in MIP (***A***, case 6, A9-CTB; ***B***, case 7, MF8-FE), MIP/PE (***C***, case 8, MF7-FE), and PEip (***D***, case 9, MF10-DY). Scale bars = 1 cm. Other abbreviations as in Fig. 1.

In six cases (cases 3–8; Table 1, Fig. 3), injections were contained fully or partly within area MIP, as defined here on the basis of myeloarchitecture. The injections in cases 3–5 targeted caudal and dorsal parts of MIP and were likely contained in the region designated dorsal part of MIP (dMIP) in our previous study (Bakola et al. 2010). The proximity of the injection sites to V6A/PEc cortex and the tissue damage that occurred as a result of the syringe penetration reduced the degree of certainty in identifying the boundaries of MIP based on myeloarchitecture in cases 3–5. Nonetheless, the connectional patterns of these cases differed in a number of ways from those of cases 1 and 2 (injections in V6Ad), and from previous reports on the connections of V6Ad (Gamberini et al. 2009) and PEc (Bakola et al. 2010). In addition, our statistical analysis showed that the pattern of projections across cases 3–7 was highly concordant (*W* = 0.724, χ^2^ = 28.96, df = 8, *p* = 3 × 10^−4^). In case 8, the injection involved area MIP but extended into area PE; data from this case are reported in Table 2 and Fig. 7 but were excluded from further analyses. Results from the MIP injections are presented in serial coronal sections of an example case (Fig. 5), and in the flat maps of Figs. 5–7.

Projections from V6Av were present after injections in MIP, albeit in smaller numbers than those observed after V6Ad injections (Table 2); however, V6Av projections to MIP originated from sites located more medial than those that projected to V6Ad (compare flat maps in Figs. 4 and 5). In contrast with cases 1 and 2, there was a marked emphasis on projections that originated from superior parietal areas (PE, PEc; Table 2), including sparse label from the anterior somatosensory cortex (areas 2 and 3a; see Figs. 5–7). Many labeled cells were located in rostral parts of the parietal convexity and in the medial bank of the intraparietal sulcus (PE, PEip); by comparison, the medial bank beyond the limits of MIP was practically devoid of label after V6Ad injections (Figs. 4 and 6). More laterally, labeled cells were more numerous in the parietal operculum and the lateral fissure (ParOp; Table 2; Fig. 5*C*), whereas the inferior parietal areas were more sparsely labeled, compared with the V6Ad injection cases. We noted a preference for VIP projections to target MIP locations in relatively ventral portions in the bank (Table 2, cases 6 and 7), reminiscent of the differences between V6Ad injections described above. Label in the lateral bank of the intraparietal sulcus (areas LIP and AIP) was weak (Fig. 5*C–E*, Table 2).

Visual projections to MIP originated from V6 (Fig. 5*A*), peripheral representations of extrastriate area V2 (Figs. 5*B*, 6, and 7), area prostriata (ProSt, Figs. 5 and 6), parts of the ventromedial cortex (Vis; Figs. 5*B* and 6), and the dorsal prelunate gyrus (DP/V4; Figs. 5–7). Consistent label in the temporal lobe was confined to areas MST and TPO, although scattered labeled cells were occasionally observed in Tpt (Fig. 5), TPOr (Figs. 6 and 7), and the parahippocampal cortex (TH/TF; Figs. 5 and 6).

Similar to the V6Ad cases, weak to moderate projections arrived from medial cortex areas (PGm, 31, 23, 24, Rs; Table 2). In cases 3 and 5, a few labeled cells were found in areas PEci/31 (Figs. 5*C* and 6). Frontal projections to MIP originated from the same complement of areas that projects to V6Ad but were denser overall (Table 2). F2 projections stemmed from nearly the entire extent of this area (e.g., Fig. 5*F*, *G*). Finally, after injections in MIP, some labeled neurons were present, perhaps surprisingly, in the depths of the posterior bank and floor of the arcuate sulcus (SEM; Figs. 5*F*, 6*B*, and 7*A*), extending to the classic FEF region on the arcuate convexity (FEF; Figs. 6 and 7), and in the periprincipal region (Figs. 5–7).

#### Injection in cortex rostral to MIP

**Figure 8.**
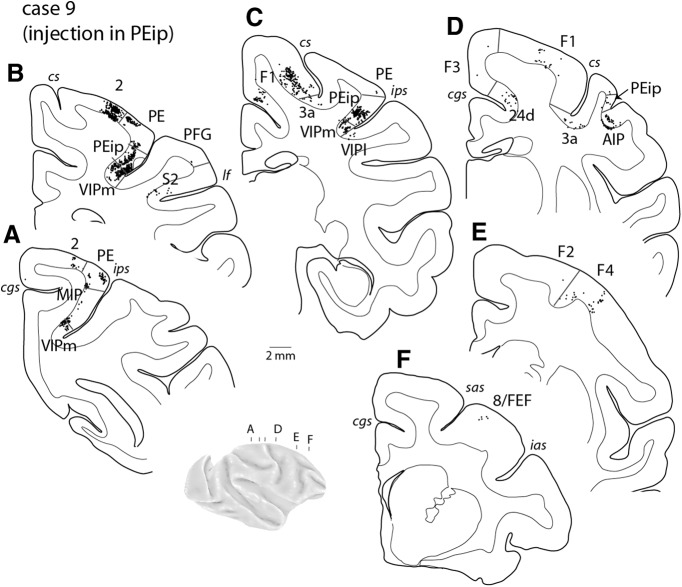
Cortical distribution of retrogradely labeled cells in case 9 (MF10-DY) with an injection in PEip, rostral to MIP. ***A–F***, Coronal sections were taken at the levels indicated on the brain figurine. *ias*, *sas,* inferior and superior limbs of the arcuate sulcus, respectively. Other abbreviations as in Fig. 1.

In one case, we placed a diamidino yellow injection in cortex rostral to the myeloarchitectural border of MIP (PEip, case 9; Fig. 3). The pattern of connections (Figs. 7 and 8 and Table 2) differed in substantial ways from the above descriptions, showing marked emphasis on input from the somatosensory areas of the anterior (area 2, Fig. 8*A*, *B*; area 3a, Fig. 8*C*, *D*) and lateral (ParOp; Fig. 7) parietal cortex and the primary motor cortex (F1; Figs. 8*C*, *D*). In the intraparietal sulcus, significant numbers of labeled cells were found in the rostral half of the medial bank, extending into lateral locations (area AIP; Fig. 8*D*) and ventrally in VIP (Fig. 8*A–C*), whereas input from medial parietal areas was limited. Unlike the cases with injections in V6Ad and MIP, projections from the premotor cortex were shifted laterally and originated mainly from ventral subdivisions F4 (Figs. 7 and 8*E* ) and F5 (Fig. 7). The differential connections with the lateral premotor cortex alone appear to be reliable anatomic indicators of caudal and rostral parts of the medial bank (see also Tanné-Gariépy et al. 2002).

#### Comparison between V6Ad and MIP

**Figure 9.**
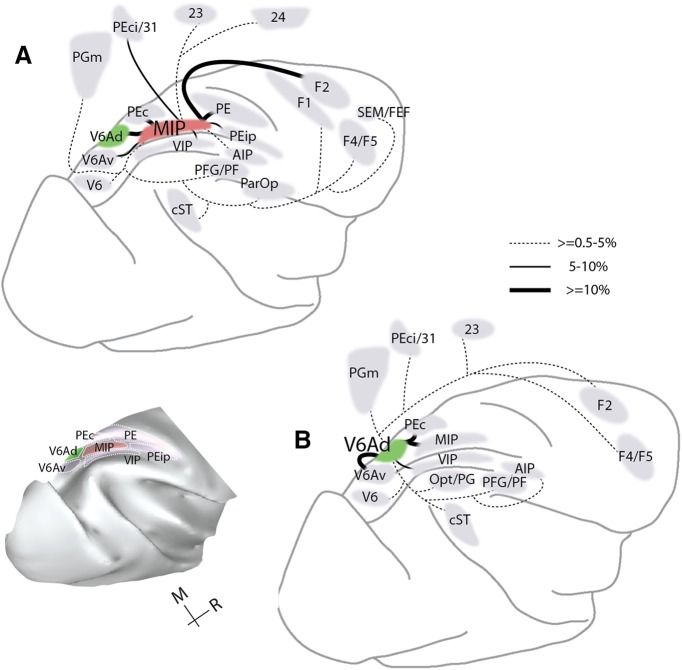
Summary view of the main projections (≥0.5%) to MIP (***A***) and V6Ad (***B***) in the present study. Parietal areas identified in the present study are shown on the brain reconstruction on the bottom left of the figure.

Areas V6Ad and MIP overlap at least in part with the territory of the physiologically defined PRR, so it is of interest to define common and distinctive anatomic features of these areas. V6Ad and MIP connect to each other (Table 2) and share a defined set of projections from the same complement of parietal association, caudal frontal, temporal, and medial areas. On average, reciprocal V6Ad-MIP and common extrinsic projections accounted for ∼90% of the total labeled neurons (82%–98% across individual cases; Table 2). Statistical analysis of the distribution of label across cases 1–7 suggests a moderate degree of concordance (*W* = 0.594, χ^2^ = 33.29, df = 8, *p* = 5 × 10^−5^). Areas that sent substantial projections to both V6Ad and MIP (≥0.5% of total average label for each area; Fig. 9) included V6, the ventral subdivision of V6A (V6Av), caudal superior parietal area PEc, medial parietal areas (PGm, PEci/31), and the fundus (area VIP) and lateral bank (area AIP) of the intraparietal sulcus. Additional projections that targeted both V6Ad and MIP originated from rostral (PFG/PF) inferior parietal areas, caudal parts of the temporal lobe in areas MST and TPO (cST; Table 2), cingulate area 23, the caudodorsal premotor area F2, and the ventral premotor cortex (subdivisions F4/F5).

Despite these commonalities, specific variations in the density and modality specificity of projections were observed (Table 2). Thus, V6Ad received overall visual association input (from inferior parietal cortex, Rozzi et al. 2008; from V6Av, Gamberini et al. 2011). In comparison, MIP received denser somatic-related input from superior parietal areas and motor input from premotor areas, the primary motor cortex (F1), and motor cingulate area 24d (Luppino et al. 1991). MIP received additional minor input from the ventromedial visual cortex (including peripheral parts of area V2) and the frontal oculomotor areas. These differences are reflected as gradual shifts in the spatial arrangement of projections to the two parietal areas; the example of the single hemisphere reconstruction of cases 1 (injection in V6Ad) and 3 (injection in MIP) of Fig. 6A illustrates this point.

We examined the laminar distribution of projection neurons to MIP and V6A by calculating the proportion of labeled neurons located in the supragranular layers (%SLN) as a percentage of the total number of labeled neurons in each projection area (Felleman and Van Essen, 1991). In this analysis, we included projections that comprised 50 or more neurons per area in at least two cases and pooled the results from different cases to avoid bias introduced by small samples (Burman et al. 2014a). For MIP, most projections fell between 32% and 58% (Table 3) and were labeled as bilaminar; in contrast, projections from cingulate area 23 originated from infragranular layers (descending type). The pooled results from the two V6A cases revealed that the projections were of a bilaminar or infragranular type. However, we have refrained from drawing any strong conclusions about the direction of information flow based solely on the retrograde labeling patterns between connected areas (Felleman and Van Essen, 1991; Rozzi et al. 2006; Hackett et al. 2014).

**Table 3.** Percentage of labeled neurons in supragranular layers after injections in V6Ad and MIP.

Area	V6Ad	MIP
V6		37
V6Av	36	53
V6Ad	^#^	52
PEci/31	46	54
PE	*	50
PEip	*	46
PEc	33	46
MIP	29	^#^
VIP	26	43
AIP	*	47
Opt/PG	30	43
PFG/PF	48	*
ParOp	*	45
cST	22	42
23	44	26
24	*	39
F1	*	58
F2	40	53
F3	*	32
F6	19	*

The table includes projections that comprised at least 50 labeled neurons projecting to an area, pooled across two cases (1, 2) with injections in V6Ad and five cases (3-7) with injections in MIP. ^#^Location of injection site; *insufficient sample size (<50 neurons per case in at least two cases).

Based on rigorous definitions of areal laminar organization, it has been suggested that projection neurons to frontal cortex stem from the upper layers of eulaminated fields, and, conversely, from progressively deeper layers of less differentiated fields (Barbas, 1986). Although a full analysis cannot be applied to the present data, due to the lack of structural classification of many of the source areas and of anterograde labeling data, there is some support for the view that structural characteristics influence connectivity. In our data. frontal projections to MIP originated in different cortical layers, depending on the laminar composition of each source area (Table 3). In particular, after injections in MIP, the proportion of labeled neurons in supragranular layers increased systematically with the architectonic differentiation of frontal motor areas, from areas 24 and F3 to the dorsal premotor and the primary motor cortex (Barbas and Pandya, 1987; Morecraft et al. 2012; Barbas and García-Cabezas, 2015), suggesting that connectional patterns vary systematically with cortical structure.

## Discussion

The focus of this study was to clarify the organization of the medial bank of the intraparietal sulcus in the macaque, on the basis of architectonic characteristics and corticocortical connections. Our starting point was the fragmented, and somewhat contradictory, available information regarding the location, extent and histologic characteristics of area MIP (Colby et al. 1988; Lewis and Van Essen, 2000a; Cavada, 2001) and its border with adjoining area V6A. These areas are often considered to overlap the physiologically defined PRR (Snyder et al. 1997), which has been studied in relation to visually guided arm movements and has become a subject of research aimed at the control of artificial limbs based on brain–computer interfaces (Andersen et al. 2014b).

### Subdivisions of the medial bank

Our observations of myelin-stained coronal sections refined prior findings in showing that approximately the caudal half of the medial bank of the intraparietal sulcus comprises two main subdivisions, which we refer to as V6Ad and MIP. The lip of the medial bank included extensions of superior parietal lobule areas (PE, PEc; Pandya and Seltzer, 1982; Morecraft et al. 2004), whereas ventrally, near the fundus, we confirmed the presence of subdivisions of area VIP, which exhibit distinct myeloarchitecture (Lewis and Van Essen, 2000a).

Although definitions of borders between association cortex areas are intrinsically criterion dependent (Rosa and Tweedale, 2005; Palmer and Rosa, 2006; Burman et al. 2008, 2014b; Gamberini et al. 2011), the present scheme seems to better conform to the expectation that cortical areas have uniform architectural appearance and connections. Nonetheless, as in other parietal areas (Bakola et al. 2010; Passarelli et al. 2017), there is the suggestion of a gradient of connections, whereby dorsal injections in the medial bank tend to reveal stronger connections with superior parietal areas PE and PEc, whereas injections in the ventral part of the medial bank reveal stronger connections with area VIP. Indeed, given the degree of commonality in connections between V6Ad and MIP, another interpretation of our data are that a large section of the medial bank of the intraparietal sulcus is formed by a single area, within which patterns of connections change in a gradual manner. Here, the relatively clear change in myeloarchitectural pattern has persuaded us to retain the subdivision of this region into V6Ad and MIP, but this is clearly a topic that deserves further study. In particular, it will be important to define which physiologic properties distinguish these subdivisions and to what extent they encompass the entirety of the PRR.

The connectivity pattern at even more rostral locations in the medial bank (PEip; Fig. 1), which is generally considered to be outside the PRR, emphasizes inputs from the anterior somatosensory, primary motor, and ventral premotor cortices, suggesting a functional zone distinct from V6A and MIP. This region is likely part of the parietal field containing large representations of the distal forelimb (Seelke et al. 2012; Rathelot et al. 2017). Rostral parts of the posterior parietal cortex, around both banks of the intraparietal sulcus, have been studied in the context of limb movements aimed at object acquisition (Gardner et al. 2007; Baumann et al. 2009), even when these movements are highly stereotypical and performed in the absence of visual information (Evangeliou et al. 2009; Nelissen and Vanduffel, 2011).

### Comparison with previous studies

Nomenclature issues aside, many of the sources of projections to MIP described here have been reported by studies in which tracer injections were placed in other areas. Among these, the most conspicuous connections are with the dorsocaudal premotor cortex (e.g., Matelli et al. 1998; Tanné-Gariépy et al. 2002). Other studies have revealed projections from the region presently defined as MIP to areas V6 and V6A (Colby et al. 1988; Shipp et al. 1998; Galletti et al. 2001; Marconi et al. 2001; Gamberini et al. 2009; Passarelli et al. 2011), the superior and inferior parietal cortices (Rozzi et al. 2006; Bakola et al. 2010, 2013), medial parietal areas PGm and 31/PEci (Morecraft et al. 2004; Passarelli et al. 2017), and area VIP (Lewis and Van Essen, 2000b). As we have shown, the above areas provide the majority of the projections to both MIP and V6Ad. The observed overall scarcity of connections with areas LIP and AIP (Table 2) is also in agreement with previous reports (Blatt et al. 1990; Lewis and Van Essen, 2000b; Borra et al. 2008).

On the other hand, reports of extrinsic connections largely or exclusively directed to the currently defined MIP region, but not adjacent areas, are rare. Among the few such instances are the selective connections with area PE (present results) the lateral parietal region (PGop, Ri; Cipolloni and Pandya, 1999; ParOp in Table 2), and with the medial and ventral premotor (in particular, area F5) cortex (Petrides and Pandya, 1984; Luppino et al. 1993; Gerbella et al. 2011). Although we did not attempt to subdivide area F5 (Belmalih et al. 2009), our data appear in agreement with those of Gerbella et al. (2011) in showing that MIP connections are restricted to the posterior subdivision, which contains a hand representation field (Raos et al. 2006).

The presence of some of the minor long-distance projections to MIP is more difficult to ascertain based on previous studies. These projections reflect only limited contributions to the overall MIP connectivity (Table 2) and, as such, might have been undetected in previous studies due to methodological factors (sensitivity of tracers, area coverage of injections, sampling), biological variability, or the existence of unidirectional pathways. For example, connections with the upper superior temporal cortex and the dorsal calcarine sulcus were either not reported by earlier tracing studies (Boussaoud et al. 1990; Seltzer and Pandya, 1991) or cannot be unequivocally inferred based on illustrations (Gattass et al. 1997). Likewise, connections with the periarcuate region have been shown in a few instances (Petrides and Pandya, 1999; Stanton et al. 2005). The projection detected in our study likely included the premotor oculomotor region (Baker et al. 2006; Savaki et al. 2015), which comprises the smooth-pursuit eye field (Stanton et al. 2005), with minor involvement of area 8/FEF on the prearcuate convexity. In New World marmoset monkeys, connections between divisions of area 8 and likely homologous dorsal parietal cortex have been consistently demonstrated (Reser et al. 2013; Burman et al. 2015). The macaque periarcuate region contains neurons with effector (eye or hand)-dependent or effector-independent discharges (Neromyliotis and Moschovakis, 2017) and constitutes a potential source of eye–hand coordination mechanisms downstream of parietal cortex (Yttri et al. 2013).

### Functional considerations relative to sensorimotor actions

The largely overlapping connectivity profiles of MIP and V6A, including input from the same territory of dorsocaudal premotor cortex, argue against strict functional segregation in the medial bank. This notion resonates with primate neurophysiological findings that show complementary activations in a wide extent of the medial intraparietal and parieto-occipital cortices related to events in peripersonal space (Colby and Duhamel, 1991; Hadjidimitrakis et al. 2011) and to different paradigms of visually guided reaching (e.g., Kalaska and Crammond, 1995; Johnson et al. 1996; Battaglia-Mayer et al. 2001; Calton et al. 2002; Fattori et al. 2005; Breveglieri et al. 2014; Rajalingham and Musallam, 2017). Anatomic and functional overlap does not appear to be unique to the medial parietal areas: similar division of labor during simple tasks occurs among neuronal populations in distinct, interconnected frontal motor regions (Russo et al. 2002; Crutcher et al. 2004) and posterior parietal-prefrontal regions (Katsuki and Constantinidis, 2012). The distribution of representations of spatial and movement parameters across different neuronal populations likely reflects the flexible strategies for problem solving (Battaglia-Mayer et al. 2003), according to available (e.g., visual) resources or the preferred effector.

A detailed comparison between the present anatomic scheme and functional localization remains unattainable, largely because of variability in areal definitions and differences in task priorities among laboratory groups. MIP is a site of convergent visual, somatic-related, and direct projections from the primary motor cortex, whereas visual input is more robust caudally, in V6A. The different weights of sensorimotor input likely exert different influences on the activity of V6A and MIP, with MIP more directly involved in representations of movement parameters (Caminiti et al. 2017) and in decision-related processes when decisions are communicated by hand movements (de Lafuente et al. 2015). Neurophysiological evidence indicates that MIP contains neurons that signal the direction of a planned movement and not the location of the visual target (Eskandar and Assad, 2002; Hamel-Pâquet et al. 2006; Kuang et al. 2016). In addition to goal-directed actions, MIP neurons display modulations to self-generated arm movements in the absence of an external trigger (Maimon and Assad, 2006). By comparison, neuronal modulations in V6A appear to reflect both spatial and reach-related information (Breveglieri et al. 2014; Hadjidimitrakis et al. 2014a). It becomes obvious, however, that any differences are subtle and that reliable attribution of regional specialization is still lacking.

Although the anatomic areas of the posterior parietal cortex have been traditionally considered specializations for effector-specific movements, recent advances in human (Hinkley et al. 2009; Leoné et al. 2014; Zhang et al. 2017) and nonhuman primate (Cooke et al. 2003; Gharbawie et al. 2011; Kaas et al. 2011) research provide new insights into the rich functional organization of different parietal fields, including charting of the temporal dynamics during actions across various cortical areas (Filimon, 2010; Vesia and Crawford, 2012; Verhagen et al. 2013; Cui, 2014; Caminiti et al. 2015; Gallivan and Culham, 2015). In this context, consistent connections of posterior temporal fields with MIP/V6A, but not with dorsal parietal areas (Bakola et al. 2010, 2013), appear to have a functional counterpart in operations relevant to covert shifts of spatial attention (Caspari et al. 2015). Likewise, studies involving nonhuman primate physiologic mapping (Taoka et al. 1998, 2000; Breveglieri et al. 2008) and connections (including subcortical input, Impieri et al. 2018), and human imaging (Abdollahi et al. 2013; Heed et al. 2016) point to a more general role for superior parietal areas PE/PEc in whole-body movements such as locomotion and climbing. Our results offer some evidence for functional modules within the medial intraparietal networks for arm and hand movements; future research guided by the present anatomic scheme may identify the full spectrum of distinct parietal contributions in the guidance of sensorimotor behavior.

## Synthesis

Reviewing Editor: Menno Witter, Norwegian University of Science and Technology Decisions are customarily a result of the Reviewing Editor and the peer reviewers coming together and discussing their recommendations until a consensus is reached. When revisions are invited, a fact-based synthesis statement explaining their decision and outlining what is needed to prepare a revision will be listed below. The following reviewer(s) agreed to reveal their identity: Helen Barbas.

The authors addressed most of the points raised in the previous reviews. It is still difficult to see architectonic borders with myelin in Fig. 1. Perhaps the authors can summarize the salient features that distinguish the different subareas in the figure legend.

The last two paragraphs of Results could be better integrated. Parts of these paragraphs belong in Discussion. The authors need not infer direction of flow of information to discuss that connections in their material are consistent with a structural principle, namely that the laminar pattern of connections depends on the structural relationship of linked pairs of cortices. This principle states that both the structure of the origin and termination matter, not just the source, as stated. The principle was formalized in a later paper, after 1986 (Cerebral Cortex, Cerebral Cortex 7:635-646, 1997), and recently reviewed in depth (Annu Rev Neurosci. 38: 269-289, 2015).

## Author Response

Editor: The authors addressed most of the points raised in the previous reviews. It is still difficult to see architectonic borders with myelin in Fig. 1. Perhaps the authors can summarize the salient features that distinguish the different subareas in the figure legend.

Authors: Descriptions of myeloarchitectonic features of the parietal subdivisions MIP, V6A, PEip, that constitute the focus of this study, have been included in the legend of Fig. 1.

Editor: The last two paragraphs of Results could be better integrated. Parts of these paragraphs belong in Discussion. The authors need not infer direction of flow of information to discuss that connections in their material are consistent with a structural principle, namely that the laminar pattern of connections depends on the structural relationship of linked pairs of cortices. This principle states that both the structure of the origin and termination matter, not just the source, as stated. The principle was formalized in a later paper, after 1986 (Cerebral Cortex, Cerebral Cortex 7:635-646, 1997), and recently reviewed in depth (Annu Rev Neurosci. 38: 269-289, 2015).

Authors: In the last part of Results, we presented data relevant to the laminar origin of projection neurons and attempted an interpretation regarding the flow of information (largely based on the Felleman &Van Essen 1991 scheme) and the structural composition of the source areas (after the original scheme by Barbas 1986). We make these data available to potentially aid future research but a more thorough discussion is beyond the design of this work as evidenced by a) scarcity of a larger body of data (Table 2); b) lack of anterograde labeling; and c) lack of detailed structural classification of other source areas, in particular, of the several subdivisions of the parietal lobe and of target areas MIP, V6A and PEip. We have edited the last paragraph of results to better convey these points.
